# Youth engagement in digital public health: from visibility to influence, accountability, and reach

**DOI:** 10.3389/fpubh.2026.1993809

**Published:** 2026-10-23

**Authors:** Alessandro Pascon Filho, Stella Goeschl, Paul Dietram, Melanie Maria Kuhrn, Swostika Thapaliya, Aferdita Bytyqi

**Affiliations:** 1 Digital Transformations for Health Laboratory, University of Geneva, Geneva, Switzerland; 2 Barretos School of Health Sciences (FACISB), Barretos, Brazil; 3 Molecular Oncology Research Center, Barretos Cancer Hospital, Barretos, Brazil; 4 Imperial College London, London, United Kingdom; 5 School of Nursing, KCMC University, Moshi, Tanzania; 6 Central Department of Public Health, Tribhuvan University, Kathmandu, Nepal

**Keywords:** accountability, digital public health, health equity, health governance, meaningful adolescent and young adult engagement, youth engagement, youth leadership

## Abstract

Digital tools increasingly shape the health of adolescents and young adults, yet these young people with the highest stakes often hold the least power over how these tools are designed, governed, and evaluated. Calls for meaningful youth engagement have become widespread, and a visible infrastructure of advisory boards, forums, and delegations has grown around it. However, participation has expanded faster than the influence it implies. In this perspective, we argue that the gap between participatory rhetoric and governance reality is largely structural: the mechanisms now in place tend to make youth engagement visible without converting it into influence over what is decided. We trace this through three tensions—visibility without influence, consultation without accountability, and global rhetoric with local exclusion—each showing how the vocabulary of participation can describe arrangements that carry little real power. Moving from consultation to leadership, we argue, calls for attention to three considerations: formal, age-appropriate decision rights for young people; a duty on institutions to document and answer youth input; and governance capacity in the settings where digital tools are actually used. We set out illustrative mechanisms and safeguards for each, to inform the digital health strategies now being drafted.

## Introduction

Digital technologies are reshaping adolescent health faster than governance has adapted. Adolescents and young adults (AYAs) are among the age groups most exposed to digital technologies worldwide (1), and the tools they encounter—health apps, algorithmically curated health information, AI chatbots, and digital mental-health services—increasingly shape the health information, recommendations, and services available to them while remaining poorly governed and poorly evaluated (1). Digital public health is understood here as the use of digital technologies—applications, platforms, information systems, artificial intelligence, and data infrastructures—to promote and protect population health; its governance is the set of rules, roles, and institutions that decide how these technologies are designed, deployed, and held to account (1, 2). In 2021, The Lancet and Financial Times Commission on Governing Health Futures 2030 stated the problem directly: digital transformations are “embedded into, and negotiated within, broader political, societal, and economic processes”, yet the young people growing up inside them hold the highest stakes and the least structural power to shape how they unfold (1).

The risks of these tools are not evenly spread. Digital mental-health tools are often promoted for low-resource settings where other routes to care are scarce, yet a systematic overview of 18 reviews found that the underlying studies came mostly from high-income countries and that none reported participants’ socioeconomic background, leaving little evidence on how these tools perform elsewhere (3). Data handling is a further problem: when researchers intercepted the traffic of 36 top-ranked apps for depression and smoking cessation, 33 transmitted user data to third parties, often without disclosing it, and detecting this depended on users’ own privacy literacy (4). A young person who uses a free app because no service is nearby has little way of knowing how well it works or where their data goes.

At the same time, we are seeing an increase in calls for meaningful youth engagement in designing, governing, and evaluating the digital tools that shape young people’s lives. The Lancet and Financial Times Commission took an explicit position: youth and youth organizations should be embedded within institutions of power, structures for meaningful youth participation should be built, and the health and wellbeing issues affecting young people should be defined by young people themselves (1). Multilateral bodies have since created mechanisms of this kind, among them the WHO Youth Council, youth delegate schemes at the World Health Assembly, and youth advisory councils across UN agencies (5). The language of co-design, co-production, and youth-led innovation now circulates across research, policy, and funding. The WHO Global Strategy on Digital Health 2020–2025 calls for person-centered, inclusive governance, but young people are not mentioned anywhere in the document (2).

This shift has produced a visible institutional infrastructure (5). Young people are increasingly invited into design workshops, appointed to advisory boards, represented at high-level meetings, and cited in reports as evidence of inclusive practice. Under the right conditions, their involvement has measurable value: an umbrella review of 99 reviews found positive effects on young people themselves and on the relevance and conduct of research (6). Yet the same literature reveals a paradox. Fewer than 1% of studies on child and adolescent health report any adolescent involvement (7), and where participation occurs it often remains concentrated in design and co-creation, while the literature continues to call for more sustained engagement through implementation and evaluation (8, 9). Participation has widened as a practice while the decision-making structures around it have changed far less.

This gap has less to do with institutional bad faith than with how these systems are put together. Governance arrangements in digital public health readily generate visible participation, and the models used to describe them makes visibility and influence hard to tell apart. In Arnstein’s ladder, placation describes participation in which citizens may advise while decision-making power remains elsewhere (10). Hart’s ladder of youth engagement places many current initiatives on its lower rungs, among them decoration and tokenism (11). Lundy’s reading of Article 12 of the Convention on the Rights of the Child adds what these ladders leave implicit: a voice counts only where an audience is obliged to hear it and where what is said carries influence over the decision. Institutions can satisfy the outward signs of consultation and still set young people’s views aside, which makes decorative participation counterproductive rather than merely inadequate (12). The stakes are particularly salient in digital health, because tools built without genuine youth influence act on the very populations excluded from their design, shaping what health information young people see and which risks an algorithm amplifies. Exclusion here is not only a question of equity; it can undermine the relevance, acceptability, safety, and uptake of the tools themselves.

This perspective piece traces that pattern through three tensions, each a place where participatory language and governance practice come apart: visibility without influence, consultation without accountability, and global rhetoric with local exclusion. We then ask what moving from consultation to leadership would take, setting out three considerations—influence, accountability, and reach—with the mechanisms and safeguards each implies. Our argument draws on the participation and governance literatures and on the authors’ own experience within these processes (5, 13, 14).

## Visibility without influence

Youth engagement has placed young people in the sightlines of major decision-making bodies. Their activities circulate in institutional and social media, and they are given a seat at the table more often than ever. But the same visibility has produced a recognizable trap. Advisory boards, delegations, and forums generate participation that can be documented and readily cited by host organizations. What they rarely confer are the formal decision rights that would turn presence into power (8, 9, 15).

An advisory board that informs and a governance body that decides both put youth in the room and both generate documented recommendations; what differs—who is obliged to act, and on what—tends to disappear in the vocabulary used to describe them. A 2026 scoping review traces the rapid growth of youth advisory boards since the UN Convention on the Rights of the Child and finds that they engage young people more often in training and data collection than in setting research direction or making decisions (15). An invitation to co-create and a seat at the governance table are described in the same institutional language, even though the two differ fundamentally in power.

Tokenistic participation is not merely ineffective; it can harm the young people involved and erode their trust (7). What is missing is not voice but influence: young people are heard, and what they say does not reliably bear on what is decided. Influence in this sense is not a matter of weighting young people’s views above anyone else’s. It is a defined, age-appropriate share in specified decisions, held on the same footing as other participants, so that what young people say has to be answered for in the outcome. The United Nations Youth Office frames meaningful engagement as recognizing young people as agents of change through inclusive partnership, intergenerational collaboration, sustained engagement, and accountability (16), each of which presupposes that decision-making power is shared. Power in these settings is not zero-sum: young people can gain a say over specific decisions without other participants losing theirs. This need not mean that every young participant holds institutional power, but a middle ground is possible. As one illustration, the second Lancet Commission on adolescent health and wellbeing appointed 10 youth commissioners who co-led workstreams, were remunerated for their contributions, and are named among the report’s authors (17).

## Consultation without accountability

Where the first tension is about who sits in the room, this one is about what happens to what is said once they are there. Participatory methods have entered the mainstream of digital and public health; consulting young people is, in many settings, now a condition of funding rather than an aspiration (18). But the near-universal adoption of participatory language has not been matched by any obligation on institutions to respond. Where no institution must act on, acknowledge, or explain its treatment of youth input, and no record is kept of whether that input changed anything, the contribution stays decorative—not through bad faith, but because no rule requires more (10, 18). A process can satisfy every participation guideline and still change nothing about what the institution does.

Consultation is now common; a duty to act on it is not. Accountability is that duty. An institution required to justify how it used youth input differs from one that merely takes it under advisement, because the requirement can turn a one-off event into a relationship that can be traced over time. In practical terms, such a duty could include two elements. First, the institution should document publicly how input was used, and give reasons when it sets that input aside. Second, this duty has to sit within a standing, mandated body rather than depend on goodwill; evidence from social-accountability practice suggests such requirements have larger and more durable effects when they are built into institutions rather than run as occasional exercises (19).

Mechanisms of this kind already exist. In health systems, “you said, we did” or “closing the loop” practices build a response obligation into a required feedback step (20). At the level of global commitments, Resolution WHA77.2—the first WHA resolution devoted to social participation—calls on Member States to implement, strengthen, and sustain regular and meaningful social participation in health-related decision-making (21). Its companion handbook translates this into guidance on linking participation to decision-making and on the legal frameworks that sustain it (22). A comparable duty could be made explicit for AYAs in digital health.

## Global rhetoric with local exclusion

The first two tensions take for granted that young people have already reached these processes. However, many never do. Calls for youth leadership in digital public health are loudest in global institutional spaces, while many of the young people most exposed to poorly governed tools live in the contexts with least access to those spaces.

Global processes, meaning the international forums where health strategies are negotiated, such as the World Health Assembly and UN youth councils, can favor young people with the institutional, financial, linguistic, and network access needed to enter them. O’Sullivan et al. (5) document the pattern: among 194 WHO Member States, the number of youth delegates at the World Health Assembly rose from 9 in 2022 to 13 in 2023, but 11 of the 13 countries represented were classified as high-income by the World Bank. Funding constraints, unequal North–South relations, displacement, poverty, and poor internet and mobile connectivity keep a wider range of young people off the global stage.

The imbalance is particularly consequential in digital health, because the harms of poorly governed tools—misinformation, algorithmic exclusion, online abuse, and unsafe data practices—can fall disproportionately on the populations absent when the rules are written. Reviewing national digital health strategies across 10 African countries with young populations, Holly et al. (14) found that the strategies overlooked young people’s specific needs and risks, and called for inclusive processes to protect their say in decisions that affect them.

Institutions commonly justify limited youth influence by raising repeated concerns: that young people lack the technical expertise governance needs, that formal decision rights raise financial and legal liability, and that no individual can represent a group as broad as “youth.” None of these is baseless; limited training, unequal power relations, and the pull toward tokenism are documented barriers to young people’s involvement (7). But left unexamined, each concern justifies itself. Where expertise is treated as the price of entry rather than something built through participation, young people stay permanently junior. Where liability keeps influence at arm’s length while visibility is still encouraged, the outcome is the very mix that breeds tokenism. Concerns about representativeness are legitimate, but they should not become a reason to exclude young people from decision-making altogether.

Closing this gap is a question of reach: governance capacity built where the consequences are felt. One delegate flown to a global forum cannot stand in for the world’s youth, and it is a design error to expect them to. As an example, the United Nations Population Fund has invested in youth-led networks such as AfriYAN and established national Youth Advisory Councils, routing influence through local and national structures rather than the global stage (5); Holly et al. (14) similarly recommend building capacity within national governments to work well with youth from many backgrounds.

## From consultation to leadership: influence, accountability, and reach

The three tensions build on one another. Being in the room is not the same as having a say in the decision; a say means little if no institution is obliged to answer; and even where influence and accountability exist, they are unevenly distributed, concentrated far from where poor governance does the most damage. What ties them together is that the arrangements now in place can make participation highly visible, and in the shared vocabulary of the field, visible participation is easily mistaken for influence, accountability, and reach.

Moving from consultation to leadership is therefore not simply a matter of consulting more often or more widely. Consultation gathers views, while leadership means young people hold defined rights over particular decisions, outputs, or evaluation criteria and can hold institutions to account for what follows. Arnstein, Hart, and existing definitions of meaningful engagement already map the rungs of participation. What draws attention here are recurring considerations for digital-health governance. Two emerge naturally from the issues raised above: influence, the decision rights discussed under the first tension, and accountability, the response duty discussed under the second. The third, which we suggest as a way forward, is reach: placing both influence and accountability in the settings where the tools are actually used, not only in global forums. Table 1 sets out, for each of the three, illustrative mechanisms and the safeguards they call for.

**Table 1:** From participatory appearance to youth leadership in digital public health governance: three considerations, illustrative operational mechanisms, and safeguards.

Consideration	Appearance → structural deficit	Illustrative operational mechanisms	Key safeguards and age-responsiveness
Influence—turn presence into power	Visibility (seats, forums, delegations) → no formal decision rights	Youth voting rights on governance bodies; youth co-chairing; agenda-setting roles; delegated control over specified outputs (e.g., co-authorship, product or content decisions); a role in budget allocation; co-ownership of evaluation criteria	Presume capacity and adapt information to age and needs; supply training and support; remunerate; avoid tokenistic single-seat roles
Accountability—answer, not just hear	Consultation → no obligation to respond	A formal response obligation; public documentation of recommendations and of accepted or rejected input with reasons; “closing-the-loop” feedback steps; monitoring indicators; periodic review of engagement quality, embedded in a standing mandate	Keep documentation proportionate; protect confidentiality; ensure feedback reaches younger participants in accessible form
Reach—place influence where tools are felt	Global representation → limited local participation capacity	National and community youth-governance structures; multilingual and offline or hybrid participation; connectivity and accessibility support; remuneration; transparent, rotating selection; targeted inclusion of marginalized groups; capacity-building close to deployment	Guard against local elite capture; rotate roles to prevent overburden; apply safeguarding appropriate to context; do not transfer responsibility without support

Mechanisms are illustrative and should be adapted to context, participants’ evolving capacities, and safeguarding requirements.

Influence of this kind must be calibrated. It should take account of safeguarding and legal considerations, and of the scale and technical complexity of the decision at hand, with training and support provided rather than assumed. Developmental stage and capacity are relevant too, although appeals to them can also be used to keep young people’s influence low, and assumptions about limited capacity often compromise participation in health research and design (23). The Committee on the Rights of the Child presumes capacity: it is not up to the child to prove it, and age alone cannot determine the weight a view receives (24). Responsibility for an inclusive process lies with those who run it (23), so calibration means adapting information to different ages and needs and supplying the support young people require to take part. The concerns institutions raise here—safeguarding, confidentiality, liability, continuity, representativeness, and the risk of overburdening young people—are real, and they call for designing influence with care. The arrangement to avoid is one that hands young people responsibility without the institutional support, remuneration, and protection that make it safe to carry.

How participation is designed also affects how much influence it carries. A scoping review of 38 health studies involving marginalized children and young people found that the most common rationale for involving them, making an output more useful or acceptable, was associated with the lowest average participation, and empowerment of a marginalized group with the highest. None of the highest-scoring studies drew on a human-rights framework, although it was the one most often cited among lower-scoring studies (23). Participation was also higher where studies combined several modes of contribution, such as drawing, photography, video, and writing, instead of relying on verbal accounts that favor the most articulate participants, and where researchers reflected on their own assumptions and on the norms that exclude young people (23). Studies that developed tools or services scored lower than exploratory ones (23), which matters for digital health, where youth involvement is still concentrated at the design stage (8, 9).

## Discussion

Taken together, the three considerations ask for what current arrangements rarely provide. None is a small adjustment, and this is why the gap can persist even when no one intends it. An advisory board and a governance body, a one-off consultation and a binding process, a delegate in Geneva and a youth network working at home all register as “engagement” in institutional reporting, though the term says nothing about where decisions are actually made.

Timing gives the argument practical weight. Decision WHA78 (22), adopted in May 2025, extended the WHO Global Strategy on Digital Health to 2027, and a successor strategy for 2028–2033 is now being drafted (25). Whether that strategy gives young people real influence, requires institutions to answer them, and extends both to the settings that need them most—or reproduces the more visible but shallower arrangements already in place—is a question youth-engagement scholarship is well placed to inform.

## Conclusion

The gap between what youth participation promises and what it delivers reflects what these arrangements actually do: they make participation visible and largely succeed at that. What they often fail to do is give young people standing, oblige institutions to answer them, or reach the settings where the consequences of digital tools are felt. Distinguishing visible participation from consequential participation is an important step toward the latter. As the successor to the Global Strategy on Digital Health is drafted, the questions worth pressing are concrete: which youth roles will carry decision rights, what institutions will be obliged to answer for, and where that capacity will sit. The strategy now in force does not mention young people at all. Whether the next one gives them a position is a reasonable test of how much has changed.

### Author positionality

This perspective is informed in part by the authors’ own experience of youth engagement and governance processes, including work associated with the DTH-Lab.

## Data Availability

The original contributions presented in the study are included in the article/supplementary material, further inquiries can be directed to the corresponding author.
